# A developmental basis for the anatomical diversity of dermis in homeostasis and wound repair

**DOI:** 10.1002/path.5589

**Published:** 2020-12-04

**Authors:** Ivy Usansky, Patrycja Jaworska, Ludovica Asti, Fiona N Kenny, Carl Hobbs, Vasiliki Sofra, Hanfei Song, Malcolm Logan, Anthony Graham, Tanya J Shaw

**Affiliations:** ^1^ Centre for Inflammation Biology & Cancer Immunology King's College London London UK; ^2^ Randall Centre for Cell & Molecular Biophysics King's College London London UK; ^3^ Wolfson Centre for Age‐Related Disease King's College London London UK; ^4^ Department of Developmental Neurobiology King's College London London UK

**Keywords:** skin, development, embryogenesis, neural crest, mesoderm, lateral plate, paraxial, tissue repair, regeneration, regional, anatomy

## Abstract

The dermis has disparate embryonic origins; abdominal dermis develops from lateral plate mesoderm, dorsal dermis from paraxial mesoderm and facial dermis from neural crest. However, the cell and molecular differences and their functional implications have not been described. We hypothesise that the embryonic origin of the dermis underpins regional characteristics of skin, including its response to wounding. We have compared abdomen, back and cheek, three anatomical sites representing the distinct embryonic tissues from which the dermis can arise, during homeostasis and wound repair using RNA sequencing, histology and fibroblast cultures. Our transcriptional analyses demonstrate differences between body sites that reflect their diverse origins. Moreover, we report histological and transcriptional variations during a wound response, including site differences in ECM composition, cell migration and proliferation, and re‐enactment of distinct developmental programmes. These findings reveal profound regional variation in the mechanisms of tissue repair. © 2020 The Authors. *The Journal of Pathology* published by John Wiley & Sons, Ltd. on behalf of The Pathological Society of Great Britain and Ireland.

## Introduction

Descriptions of the skin typically refer to the whole organ (epidermis, dermis and hypodermis from head to toe) as one entity. Skin is, however, specialised to fulfil functions specific to each part of the body. For example, dermal thickness, vascularisation, appendage density and abundance of adipose tissue are all features that vary with location. Furthermore, there are differences between skin regions with respect to wound healing and disease states [1, 2, 3].

The dermis is instructive during embryonic development, specifying the patterning of epidermal appendages such as hair follicles and glands [4]; for this reason, our attention is focused on this layer. The dermis is a dense, irregular, soft connective tissue, that is vascularised, innervated and provides important immunological defence [1]. The bulk of the dermal ECM, which dictates the physical features of the skin such as the flexibility and resilience in health and stiffness in scars, is largely the product of dermal fibroblasts, the main structural support cell of the tissue. Importantly, fibroblasts are a heterogeneous population of cells [5]. There is ‘local heterogeneity’, or in other words, at a single skin site there are multiple fibroblast lineages that are spatially and functionally distinct. For example, papillary fibroblasts located in the upper dermis are less scar‐prone compared with reticular (deep) fibroblasts that contribute to early repair and scar formation [6, 7]. Moreover, a ‘regional heterogeneity’ has been discovered in cultured fibroblasts harvested from sites across the body, with cells having specific *Hox* gene expression patterns encoding their positional identity within the body plan [8, 9]. However, one crucial aspect of regional heterogeneity that has been largely overlooked is the developmental origin of the tissue; the facial dermis is uniquely derived from the neural crest, whereas the dermis of the rest of the body is derived from mesoderm (abdomen/limbs from the lateral plate mesoderm, back from somitic mesoderm) [1]. We hypothesise that the embryonic origin of the dermis has a significant effect on adult tissue biology, including its response to wounding.

To test the hypothesis that dermis embryonic origin underpins regional characteristics of skin, we carried out RNA sequencing (RNA‐seq) of whole adult skin, during homeostasis and wound repair, comparing three anatomical sites representing the distinct embryonic tissues from which the dermis can arise. Our findings define transcriptional and functional differences between sites and demonstrate profound implications for wound repair.

## Materials and methods

### Animals

All experiments were conducted according to UK Home Office regulations. CD‐1 mice were obtained from colonies at King's College London. For wound experiments, age‐matched mice (7 weeks of age) were anaesthetised and given an analgesic, then shaved and subjected to one (cheek) or two (abdomen/back) 2 mm diameter full‐thickness excisional wounds at a single site using biopsy punches. Cheek wounds were made over the zygomatic arch in the region between the eye and the ear. This approach wounded external skin only, to the depth of the deep fascia, and did not involve the oral mucosa or affect observable feeding behaviour.

### Fibroblast cell cultures

Skin was harvested from multiple sites of 3‐week‐old mice. Unless indicated otherwise, all culture solutions were from Sigma‐Aldrich UK/Merck (Gillingham, UK). All cultures were in high‐glucose DMEM +10% FBS (Hyclone, Thermo Fisher Scientific, Loughborough, UK) with 4 mm l‐glutamine, 1 mm pyruvate and penicillin (100 U/ml)/streptomycin (0.1 mg/ml). For explants, whole skin was diced (<1 mm^2^ fragments) and allowed to adhere (30 min, 37 °C) before adding medium and allowing outgrowth. For enzymatic digestion, tissue was de‐epithelialised using 1% Dispase II (Merck); the dermis was then processed using a Human Skin Dissociation kit (Milenyi Biotec, Bisley, UK). Phase‐contrast images were captured using an EVOS Cell Imaging System (Life Technologies, Loughborough, UK).

### RNA extractions

All RNA extractions were performed using the Total RNA RNeasy Mini Kit (Qiagen, Manchester, UK). Tissue was harvested using a biopsy punch (2 mm diameter) and immediately frozen in liquid nitrogen. Subsequently, tissues were thawed in lysis buffer and dissociated using a QIAshredder (Qiagen). For validation of homeostasis gene expression, new (paired) skin samples were collected and fresh RNA extracted. Validation of wound results was carried out on the same RNA as was sequenced. For fibroblast RNA collection, early passage cells (<P3) were scraped directly into lysis buffer.

### RNA sequencing

Total RNA was provided to BGI (Yantian District, Shenzhen, PR China) for RNA quality control (Agilent Bioanalyzer 2100), library construction and sequencing using their BGISEQ‐500 platform (pair‐end reads of 100 bp). Complete data have been deposited in the NCBI Gene Expression Omnibus database, accession number GSE151850.

### Bioinformatics

Initial data analysis was handled by BGI. Data were filtered, then mapped to the reference mouse genome GRCm38/mm10 (https://www.ncbi.nlm.nih.gov/assembly/GCF_000001635.20/ accessed October 2019) using HISAT2 (V2.0.4)[10]. Clean reads were mapped using Bowtie2 (v2.2.5)[11] and then calculated using RSEM software (v1.2.12)[12]. Differentially expressed genes were calculated using DEseq2, as described previously [13]. Differentially expressed genes were further analysed in‐house; principal component analysis and hierarchical clustering were performed using MeV software [14], Venn diagrams generated using Venny 2.1 (https://bioinfogp.cnb.csic.es/tools/venny/), and graphs plotted with Graphpad Prism 8 (San Diego, CA, USA). An enrichment analysis was performed using g:Profiler [15].

### Reverse transcription and quantitative PCR

RNA was reverse transcribed using RNase H‐minus MMLV reverse transcriptase (ThermoScientific/Life Technologies). Diluted cDNA (1:10) was amplified in a Qiagen Rotor‐Gene Q using Bioline Sybr‐Green SensiMix (Meridian Biosciences, Memphis, TN, USA) with gene‐specific primers (Merck; Table 1). Cycling conditions were: 95 °C for 10 min, followed by a 45‐cycle run (95 °C for 15 s, 60 °C for 30 s, 72 °C for 30 s). Transcript abundance was quantified against a standard curve of pooled mouse cDNA (six × five‐fold dilution series) and normalised to *Fn1* expression (for wound tissue experiments) or *Actb* (for homeostasis tissue and cell culture), which were similarly quantified against a curve.

**Table 1:** Primer sequences used for RT‐qPCR.

Gene symbol	Forward oligo sequence (5'‐3')	Reverse oligo sequence (5'‐3')
*Tbx5*	AACAGTAGCAGCTAGCTTGGG	TGCCCGCGCGAGGTT
*Zic1*	AGCGACAAGCCCTACCTTTG	TGAGCCCTGAGAAGAGGACT
*Hoxb6*	TGTTCGGAGAGACCGAGGAG	AGGGTCTGGTAGCGTGTGTA
*Spp1*	TCTGATGAGACCGTCACTGC	AGGTCCTCATCTGTGGCATC
*Tgfb1*	AAGTGTGGAGCAACATGTGGAA	CAAGAGCAGTGAGCGCTGAA
*Tnc*	CAGTCAGGGCGTTAACTGGT	TGGAATTAATGCCCGCTTAC
*Timp1*	ATAGCTTCCAGTAAGGCCTGTAGCT	GTACCGGATATCTGCGGCATT
*Fn*	AGACAATGCCGTGGTCCTAACA	GAGTTGGCGGTGATATCAGAAGA
*Actb*	GCTACAGCTTCACCACCACAG	GGTCTTTACGGATGTCAACGTC

### Cell‐derived matrices

Cell‐derived matrices were established by plating fibroblasts at a confluent density on gelatin‐coated and cross‐linked coverslips (~1 × 10^5^/well in a 24‐well plate) as described previously [16]. After cells adhered overnight, the medium was supplemented with 50 μg/ml ascorbic acid, and the culture maintained for 8 days, with medium changed every 48 h. Matrices inclusive of cells were fixed (4% paraformaldehyde, PFA) and immunostained for fibronectin.

### Immunostaining

For Ki67 analysis, immediately after tissue enzymatic digestion (passage 0), fibroblasts were plated onto gelatin‐coated coverslips. After 3 days, cells were fixed (4% PFA) and immunostained. Coverslips were washed and cells permeabilised with PBS + 1% Triton X‐100, blocked using 0.3 m glycine, then washed again. Samples were then blocked in 10% goat serum + 0.5% BSA (Merck) before adding the primary antibody (Abcam, Cambridge, UK; ab16667 diluted 1:500) for overnight incubation. Cell‐derived matrix samples were blocked in 4% BSA, before adding the primary antibody (Abcam; ab23750 diluted 1:1000). The following day, coverslips were washed with PBS, then secondary antibody (goat anti‐rabbit Alexa Fluor 546, Life Technologies; A‐11010 diluted 1:750) was added for 1 h at room temperature, followed by DAPI (Merck; diluted to 1 μg/ml) for 10 min. Coverslips were rinsed with PBS then deionised H_2_O prior to mounting with DAKO Fluorescence Mounting Medium (Agilent, Santa Clara, CA, USA). For Ki67, images were acquired using an Axioplan II (Zeiss, Cambridge, UK). Cell‐derived matrices were imaged using an LSM880 inverted confocal microscope (Zeiss). For Ki67 analysis, one randomly selected field of view from three coverslips/site representing three independent cell/mouse isolations were assessed (Ki67‐positive/total nuclei). The percentage of Ki67‐positive cells was plotted and compared using the non‐parametric Friedman test and Dunn's multiple comparisons test.

### Histology and collagen assays

Skin samples were fixed in 4% PFA, dehydrated in ethanols and embedded in paraffin according to standard protocols. Tissue sections cut at 5 μm were stained with H&E or Haematoxylin Van Gieson (HVG). Images were captured using a Zeiss Axioplan II and processed using FIJI [17] and Adobe Illustrator (San Jose, CA, USA). For second harmonic generation imaging, rehydrated paraffin‐embedded sections were mounted using DAKO Fluorescence Mounting Medium and imaged using a Zeiss 7MP multiphoton microscope.

The Sircol Collagen Assay (Biocolor, Carrickfergus, UK) was used according to the manufacturer's instructions to analyse 4 mm diameter biopsy punches (three mice, three sites each) for pepsin‐soluble (0.1 mg/ml pepsin in 0.5 m acetic acid at 4 °C overnight), insoluble and total collagen.

## Results

### A transcriptional memory of developmental origin in homeostasis

**Figure 1:**
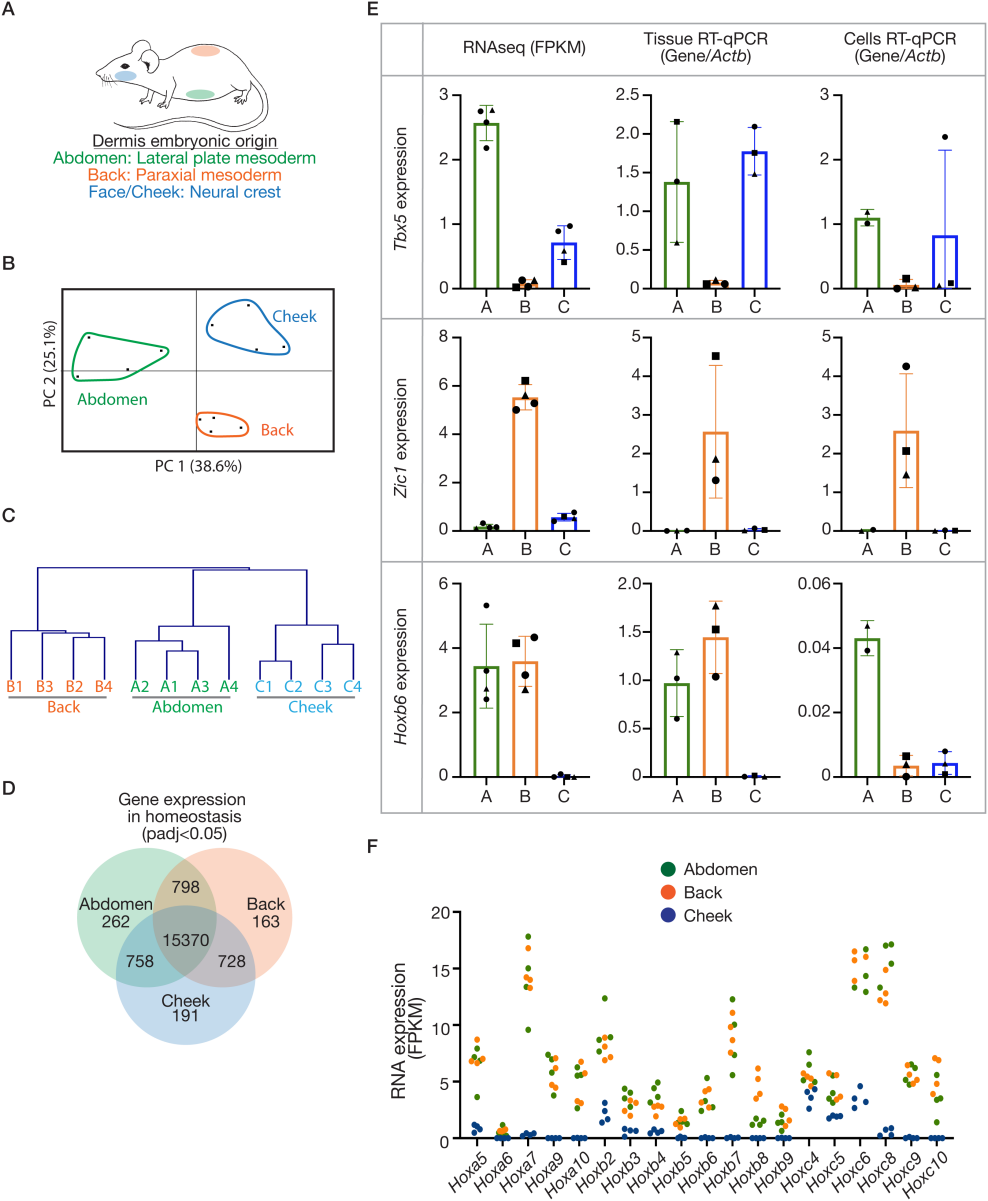
Anatomical variations in adult mouse skin in homeostasis. (A) Schematic of the embryonic origin of the dermis at different sites. (B) Principal component analysis and (C) hierarchical clustering based on gene expression of all genes significantly different between any site (padj < 0.05). (D) Venn diagram illustrating gene numbers with global or site‐specific expression (cut‐off: padj < 0.05. unpaired, two‐tailed, non‐parametric *t*‐tests). (E) RNA expression of ‘developmental genes’ relevant to the different sites, determined by RNA‐seq [fragments per kilobase per million mapped reads (FPKM), left], RT‐qPCR on independent tissue (middle) and fibroblasts cultured from different sites (right). RT‐qPCR plots display relative quantification, where concentrations were determined against a standard curve, and results normalised to *Actb*. (F) RNA expression (FPKM) of *Hox* genes with significantly less expression in the cheek sample (padj < 0.05) consistent with a neural crest origin for this tissue. Plotted points are skin/wound isolations from three or four mice per anatomical site. Bars/error bars = mean ± SD. Abbreviations and colour‐coding: A, abdomen (lateral plate mesoderm‐derived; green); B, back (paraxial mesoderm‐derived; orange); C, cheek (craniofacial, neural crest‐derived; blue).

To investigate the transcriptional differences in skin from anatomical sites with distinct embryonic origins, we performed RNA‐seq on full‐thickness skin biopsies from the abdomen (lateral plate mesoderm‐derived), back (paraxial mesoderm‐derived) and cheek (craniofacial, neural crest‐derived) (Figure 1A). A comparison of the signatures across sites revealed that, of the 18 271 genes detected (full dataset accessible at: GSE151850), approximately 15% (2901) differed significantly at a threshold of adjusted *P* value (padj) < 0.05. A principal component analysis and hierarchical clustering of these genes (Figure 1B,C) revealed a clear separation of samples by site.

Next, the transcriptomes of the three sites were overlaid to uncover site‐specific signatures (Figure 1D, supplementary material, Table S1). To question whether these signatures include a memory of the embryonic origins of the tissue, gene ontology (GO) enrichment analysis was performed. This revealed that the GO term ‘developmental process’ (GO: 0032502) was significantly enriched in both the back and cheek signatures (supplementary material, Table S2), indicating that adult skin does retain features of its variable embryonic precursors. An example for each site was validated using reverse transcription and quantitative PCR (RT‐qPCR) on independent tissue RNA (Figure 1E). *Tbx5*, whose expression is associated with the upper limb lateral plate mesoderm during development [18], was most highly expressed in the abdomen samples, and additionally in the cheek but not the back. *Zic1*, which is expressed in developing somites and their derivatives including dermis [19], was confirmed to be uniquely expressed in the back skin. Finally, *Hoxb6* was found to be absent in cheek samples, but present in abdomen and back tissue, which is consistent with the well‐established role for nested *Hox* gene expression to pattern the anterior–posterior axis [20, 21]. Furthermore, many genes in the *HoxA*, *HoxB* and *HoxC* clusters were notably absent in the cheek samples, as would be predicted (Figure 1F). We also investigated whether the expression differences persisted in primary dermal fibroblast cultures; *Tbx5* and *Zic1* expression was consistent, however, *Hoxb6* was not maintained in back fibroblast cultures (Figure 1E). These findings establish that there are significant anatomical variations in the transcriptome of adult skin, and these differences include genes implicated in the distinct embryonic development of the tissue.

### Anatomical variation in the response to wounding

The functional implications of skin differences across body sites were then investigated in the context of wound repair. Punch biopsy wounds at the three anatomical sites were collected on day 3 post‐injury. At the time of tissue collection, there were marked gross and histological differences (supplementary material, Figure S1). For example, more persistent scabs were observed in the cheek wounds than at the other two sites. There were also differences in the extent of re‐epithelialisation, with abdomen and cheek wounds having epithelial fronts significantly advanced over the wound beds compared with back wounds, which at this day 3 timepoint showed little advancement of the epidermis over the granulation tissue, and rather appeared to have reduced the wound bed size primarily by contraction (supplementary material, Figure S1).

**Figure 2:**
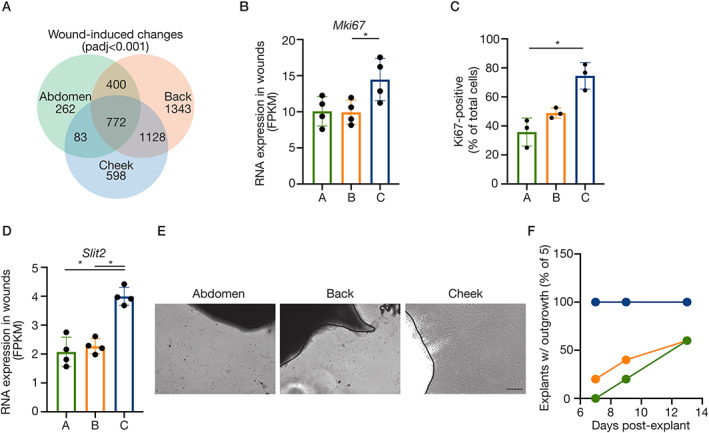
Distinct wound responses across anatomical sites. (A) Venn diagram illustrating wound‐induced gene expression changes by site (cut‐off: padj < 0.001, paired, two‐tailed, non‐parametric *t*‐tests). (B) RNA expression (FPKM) of *Mki67*. (C) Ki67 immunostaining in primary fibroblasts (passage 0) from different anatomical sites. Individual points represent mean Ki67‐positive nuclei (counted in >2 fields of view per cell line) as a percentage of total nuclei (DAPI‐stained), three (paired, i.e. mouse‐matched) independent cell isolations are plotted (*p < 0.05, non‐parametric Friedman test with Dunn's multiple comparison). (D) RNA expression (FPKM) of *Slit2*. (E) Phase‐contrast images of cellular outgrowth from dermis explants (day 9). Scale: 100 μm. (F) Time‐course of fibroblast outgrowth from dermis explants, plotting percentage of explants with visible emigrating cells. (B,D) Plotted points are skin/wound isolations from four mice per anatomical site. Bars/error bars = mean ± SD. Abbreviations and colour‐coding: A, abdomen (lateral plate mesoderm‐derived; green); B, back (paraxial mesoderm‐derived; orange); C, cheek (craniofacial, neural crest‐derived; blue).

To comprehensively characterise the site‐specific mechanisms of tissue repair, we extended our RNA‐seq to include a two‐way comparison of the day 3 wounds to paired unwounded control tissue at the three sites (*n* = 4). Dramatic changes in the transcriptome upon wounding were observed. Of approximately 17 700 total genes, 1517 (abdomen), 3643 (back) and 2572 (cheek) genes were significantly altered (up‐ and downregulated) in wound tissue (padj < 0.001), equating to 10–15%. As expected, there were many wound‐induced changes shared across the three sites, 772 in total (Figure 2A, supplementary material, Table S3). For example, there was dramatic, consistent induction of osteopontin (*Spp1*), tenascin C (*Tnc*), *Tgfb1* and *Timp1*, which were confirmed using RT‐qPCR (supplementary material, Figure S1). However, a remarkable number of wound‐induced genes varied with site at this single day 3 timepoint (supplementary material, Table S3), and it was these site‐specific wound responses that we aimed to define.

### Anatomical variations in proliferation, migration and ECM properties

The site‐specific transcriptional programmes induced during wound repair revealed differences in cell proliferation, migration and ECM that were further explored. Cheek wounds had significantly higher expression of the proliferation‐associated gene *Mki67* (Figure 2B) with a similar trend for cheek skin in homeostasis, and this was consistent for numerous proliferation markers (e.g. *Top2a*, *Mcm2*, *Mybl2*, *Ccnb1*)[22]. To confirm these results, fibroblasts were cultured from dermis of the three sites and analysed for Ki67 expression. Cheek fibroblasts had a greater proportion of positive cells compared with cultured abdomen and back fibroblasts (Figure 2C).

Enrichment analyses of the site‐specific wound‐induced signatures highlighted ‘regulation of cell motility’ in the genes uniquely upregulated in cheek wounds (padj = 0.018, 22 genes). A prominent example is *Slit2*, which was induced only in cheek wounds (Figure 2D) and is associated particularly with neural crest cell migration [23]. *In vitro* experiments again validated the gene expression data; the consistency of fibroblast outgrowth from dermis explants varied by site, with cheek fibroblast outgrowth more reliable versus abdomen and back (Figure 2E,F).

Particularly striking was the variation in ECM between sites in both the control and wound contexts. In homeostasis, the ECM GO category (GO: 0031012; extracellular matrix) was significantly enriched in both back and cheek, with 93 of 484 genes assigned to this term having significant differences across sites. Hierarchical clustering of the samples based on these genes confirmed that skin sites are divergent with respect to this parameter (supplementary material, Figure S2) even prior to wounding. When comparing the wound transcriptomes and the wound‐induced signatures, again there was a significant enrichment for ECM terms (supplementary material, Table S4).

**Figure 3.**
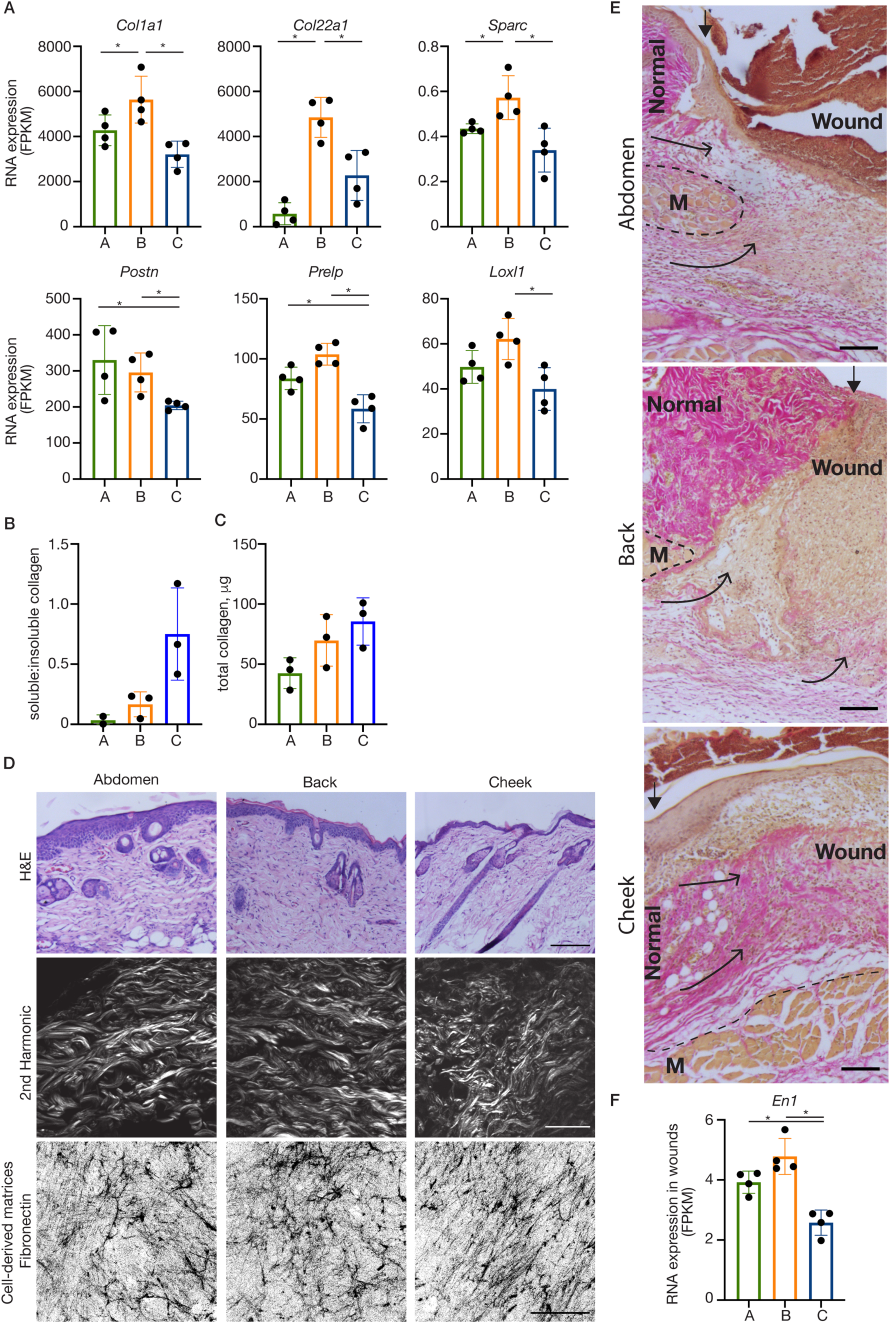
Anatomical variations in dermal ECM. (A) RNA expression (FPKM) of differentially expressed ECM genes with back‐ (top row) and cheek‐ (bottom row) specific expression patterns. Plotted points are skin/wound isolations from four mice per anatomical site. *padj < 0.05. (B) The ratio of soluble:insoluble collagen and (C) total collagen determined using Sircol assays on 4 mm biopsy punches of skin from different anatomical sites. Plotted points represent paired (i.e. mouse‐matched) independent skin isolations from two or three mice. (D) Histological analyses (H&E, top row) and second harmonic generation imaging (middle row) of dermis illustrate site‐specific characteristics (e.g. bundle thickness, density, orientation) of the collagenous ECM. Scale (H&E): 200 μm; (second harmonic): 50 μm. (Bottom row) Representative examples of maximum projection confocal images of cell‐derived matrices (immunostained for fibronectin) from fibroblasts established from dermis of different anatomical sites. Scale: 50 μm. (E) HVG histology illustrates differing tissue responses to damage, particularly the contribution of collagenous matrix (bright pink with HVG stain) from the fascia deep to the panniculus carnosus muscle (M). Scale bars: 100 μm. Arrowheads (filled): watershed between normal dermis and the wound bed; arrows (open heads) illustrate the contribution of tissue layers to the wound bed. (F). RNA expression (FPKM) of Engrailed *(En)‐1*. Bars/error bars = mean ± SD. Abbreviations and colour‐coding: A, abdomen (lateral plate mesoderm‐derived; green); B, back (paraxial mesoderm‐derived; orange); C, cheek (craniofacial, neural crest‐derived; blue).

Due to the importance of the collagen matrix in skin function, wound repair and scarring, we focussed on contributors to collagen fibrillogenesis, organisation and stabilisation, and noted that back skin had higher levels of numerous genes involved in these processes, whereas cheek had less. For example, in homeostasis, back skin had higher levels of *Col1a1*, as well as *Col22a1* and *Sparc* (both involved in the integrity and stability of the ECM), whereas cheek skin has less *Postn*, *Prelp* (both involved in fibrillogenesis) and *Loxl1* (role in collagen cross‐linking)(Figure 3A). Site‐specific differences in the collagen matrix in normal adult skin were further investigated by analysing the quantities of pepsin/acetic acid‐soluble versus ‐insoluble collagen, which evaluates matrix stability [24]. Using a colorimetric Sircol assay on 4 mm diameter biopsy punches, we observed that the ratio of soluble to insoluble collagen was higher in the cheek samples, although total collagen protein seemed consistent across sites (Figure 3B,C). This was corroborated by our observation that skin from the three sites had differential sensitivities to enzymatic digestion, with skin of the back more difficult to enzymatically digest (supplementary material, Figure S2).

Differences in the ECM architectures were also apparent histologically (Figure 3D). Analysis of healthy unwounded adult skin using H&E staining and second harmonic generation imaging, which reveals the collagen architecture [25], showed the ECM from the face to be more thin and wispy compared with the thicker bundles of the abdomen and back. The organisation was also distinct, with fibres in the cheek orientated largely in the same direction, whereas the abdomen and back had a range of orientations. When primary fibroblasts in culture were supplemented with ascorbic acid to stimulate production of cell‐derived matrices [16], similar site‐specific ECM characteristics were observed (Figure 3D).

In repairing wounds, the time course, characteristics and mechanisms of ECM reconstitution within the wound bed also had notable differences (Figure 3E). Histologically, the cheek had the most extensive ECM in the new tissue at this early timepoint (evident in HVG histology). In back wounds, the delineation between the margin and the wound bed granulation tissue was conspicuous due to the relatively sparse ECM in the new tissue. Additionally, we observed differences in the mechanism of wound repopulation. Mobilisation of deep connective tissue in repairing abdominal and back wounds could be inferred, whereas in the cheek wounds, the bulk of the repopulating tissue appears to be recruited from the wound margins superficial to the muscle layer, and accordingly may have different properties. This is consistent with the discovery that deep fascia makes a significant contribution to repairing dorsal wounds [26], and is supported by the RNA‐seq data. Specifically, Engrailed1 (*En1*)‐positive fibroblasts are thought to constitute the majority of this cell type in subcutaneous fascia [26], and to be largely responsible for scarring in the wound response [7]; there was significantly lower expression in cheek wounds compared with the other two sites (Figure 3F).

### Dermal repair re‐enacts embryonic gene expression programmes

**Figure 4:**
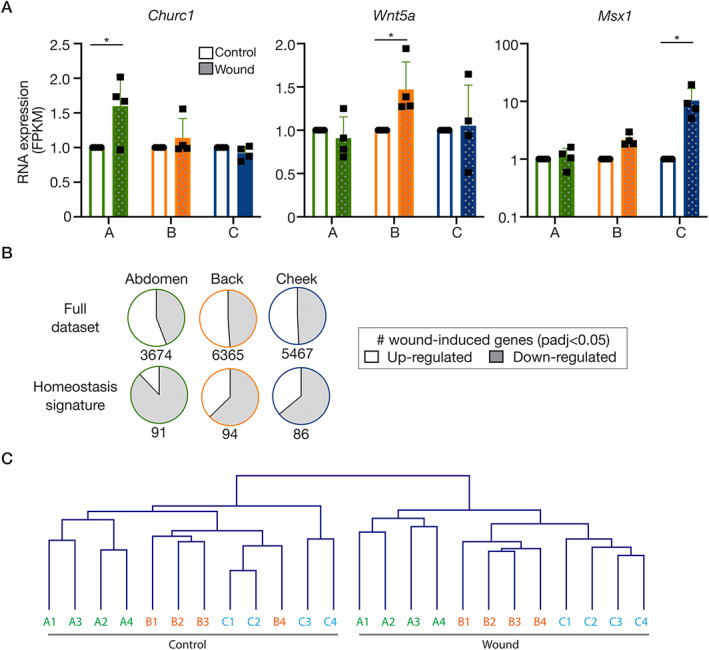
Dermal repair re‐enacts embryonic gene expression programmes. (A) RNA expression (FPKM) of ‘developmental genes’ differentially induced across sites. (B) Pie charts illustrating the directionality of change of all genes significantly altered in wounds versus unwounded samples (padj < 0.05; upregulated: open; downregulated: shaded). Top row: full dataset; bottom row: site signatures in homeostasis from Figure 1. (C) Hierarchical clustering of the samples based on the entire RNA‐seq dataset, showing that the sites remain distinct during a wound response. Abbreviations and colour‐coding: A, abdomen (lateral plate mesoderm‐derived; green); B, back (paraxial mesoderm‐derived; orange); C, cheek (craniofacial, neural crest‐derived; blue).

Having noted the embryonic memory of skin in homeostasis, we interrogated our data to question whether repairing dermis also expresses and/or re‐expresses developmental gene expression programmes. As with our analysis of the homeostasis signatures, the GO term ‘developmental process’ (GO: 0009888) served as a starting point (supplementary material, Table S2). Comparable with the unwounded skin, the wound bed site signatures contained many relevant genes involved in the tissue development. For example, abdomen wounds expressed more *Hand2*, *Tbx3*, *Tbx5*; back wounds expressed more *Gpc3*, *Sfrp4*, *Wnt2*; cheek wounds expressed more *Foxl2*, *Nkx2‐5*, *Hoxd3*. When considering the genes that are wound‐induced (i.e. significantly different from the site‐matched unwounded control), additional relevant developmental genes were discovered to be participating in the repair process. Specifically, *Churc1* (involved in mesoderm specification [27]) was uniquely induced in the abdomen wounds, *Wnt5a* (associated with paraxial mesoderm development [28]) was only significantly upregulated in back wounds and *Msx1* (important in neural crest development [29]) was only induced in the cheek (Figure 4A). Finally, we analysed if and how the homeostasis signatures for the different sites (Figure 1, supplementary material, Table S1), including features of embryonic memory, change during wound repair. We observed that more than one‐third of signature genes were altered in the wound context and, remarkably, 89% (abdomen), 63% (back) and 64% (cheek) of those changes were downregulated (Figure 4B, supplementary material, Table S5), whereas the global wound‐induced changes were essentially evenly split between up‐ and downregulated. Taken together, these findings strongly support the hypothesis that repairing tissue is returning to an embryonic‐like (less differentiated) state. Yet, importantly, hierarchical clustering of the full dataset informs us that this does not result in the samples/sites becoming more similar to one another and indeed the sites remain transcriptionally distinct, consistent with embryonic origin (Figure 4C).

## Discussion

There are remarkable variations in the skin depending on the anatomical site, from hair follicle and blood vessel density to disease susceptibility; however, there is scant cellular and molecular information about the extent of the differences and the functional implications. We hypothesise that the embryonic origin of the dermis underpins regional characteristics of skin. A focus on the dermal layer is warranted by its integral contribution to skin functionality: it provides instructive cues to the epidermis during embryonic development, dictating the patterning of epidermal appendages [4]; and its ECM determines the physical features of the skin, such as flexibility in health and stiffness in scars.

We have compared three anatomical sites representing the distinct embryonic tissues from which the dermis can arise: abdomen (lateral plate mesoderm‐derived), back (paraxial mesoderm‐derived) and cheek (craniofacial, neural crest‐derived), during homeostasis and wound repair using RNA‐seq, histology and fibroblast cultures. Our analysis comparing the transcriptomes of skin in homeostasis revealed approximately 15% variation, which demonstrates both remarkable convergences, yet considerable persistent dissimilarity. Within the site‐specific signatures there were numerous examples of genes representing the distinct embryonic development of the tissue at that site, including *Tbx5* (abdomen), *Zic1* (back) and a broad lack of *Hox* gene expression (cheek), establishing that adult skin retains a memory of its embryonic history. Although there are significant reports that distinct fibroblast lineages may lose some features of their phenotype in cell culture [30, 31], we found that cells cultured from the dermis can retain key markers of the different sites. We suggest that the examples where expression did not persist (e.g. *Hoxb6* in back fibroblasts) can be informative about cell plasticity and will improve our understanding of cell culture models.

We next investigated the functional importance of the anatomical variations of skin in the context of wound repair, which is generally accepted to vary with site, although we do not yet understand how and why. We analysed the transcriptome of day 3 wound tissue, a timepoint selected to focus on the fibroblasts repopulating the wound bed [32]. As anticipated from other transcriptional profiling experiments of wound repair [33, 34], we observed dramatic changes in the transcriptome upon wounding, with 10–15% changing compared with the unwounded tissue. There was a large number of wound‐induced gene expression changes common to all three sites, but unexpectedly, more genes had site‐specific wound responses. Comparisons between the wound beds and between the wound‐induced genes revealed site‐specific signatures suggestive of variations in cell proliferation, cell migration and ECM composition. A future analysis of the full wound healing time course will be informative as to whether these reflect qualitative differences in the healing response across sites or altered dynamics.

The more reliable emigration of the cheek cells from explants in culture, which suggests a greater migratory capacity, is consistent with their developmental origin; neural crest cells are an inherently migratory cell population that emerge from the neural tube during early development and then disperse to sites throughout the embryo [35, 36]. The ECM was also strikingly different between sites. Collectively, the RNA‐seq data, the measurements of soluble and insoluble collagen and the enzymatic digestibility of the tissue indicate a more stable ECM in the back and less stable in the cheek. The architecture of the ECM, which encompasses both composition and organisation, is profoundly influential on resident cell behaviour [37], thus we predict that these extensive differences will contribute to anatomical variations in skin functionality.

Site comparisons of the wound bed histology highlighted interesting differences in the mode of repopulating the lost tissue that are predicted to affect the ECM and scarring outcomes. We observed deep fascia contributing to the new tissue within abdominal and dorsal wounds, as was recently described [26], whereas tissue repopulating the cheek wound beds seems to arise from wound‐adjacent dermis superficial to the muscle layer. Consistent with the bulk of deep fascial fibroblasts being *En1*‐positive [26], we also detected more *En1* expression in the abdominal and back wounds compared with the cheek. This is anticipated to be of functional significant, as *En1*‐positive fibroblasts contribute significantly to scarring [7]. These observations may in part reflect regional variations in tissue architecture that have been described for facial skin (e.g. insertion of muscles of facial expression in the dermis, close proximity and association of the dermis with the underlying periosteum and bone, presence of deep adventitia [38, 39]), but this is the first indication that these structural differences may have important consequences to wound healing.

For many years, wound repair has been described to recapitulate development; however, this statement has referred to the wounded epithelium, likening its repair to developmental epithelial fusion events such as neural tube and palate closures [40]. It is not yet known whether this is also true of the dermis, but this has the potential to at least partially explain anatomical differences in *in vivo* wound repair. Our data excitingly revealed site‐specific wound‐induced expression of numerous relevant developmental genes (e.g. *Churc1* in abdominal wounds, *Wnt5a* in back wounds and *Msx1* in cheek wounds) and also uncovered that a large majority of the genes making up the site‐specific signatures in homeostasis are downregulated during wound repair, yet remain distinct. Together, these findings indicate that the dermis partly re‐enacts embryonic programmes during its repair.

This work demonstrates that adult skin, in homeostasis and during a wound response, varies significantly with anatomical site. Many of the transcriptional differences we describe echo the distinct developmental histories of the dermal fibroblasts. Although some distinct mechanisms of development of the ventral, dorsal and cranial dermis have been described [41, 42, 43], additional research into dermis development, its anatomical variations and its implications for complex tissue characteristics, such as immune composition [44] and microbiome [45], is required. Similarly, it will be valuable to determine whether the neural crest origin of connective tissue in the oral cavity influences its special healing properties [46, 47, 48].

Our findings have broad practical implications for how we study and understand skin biology, wound healing and dermatological diseases. For example, the site of tissue origin of dermal fibroblasts derived for cell culture is very rarely specified; also, mouse models with dorsal wounds are most common and practical but may not be representative of all skin repair processes. In addition to informing refinement of *in vitro* and *in vivo* experimental wound models, this work has the potential to add a new dimension to the emerging knowledge about fibroblast heterogeneity, improve our understanding of site‐specific skin diseases, as well as uncover information about cells and their scaffolds that can be harnessed for improved wound treatments and regenerative medicine.

## Author contributions statement

IU, PJ, LA, FNK, CH, VS and HS performed laboratory work and data analysis. ML and AG helped conceive/design the study, participated in data analysis, and critically revised the manuscript. TJS helped conceive/design the study, coordinated and carried out laboratory work and drafted the manuscript. All authors gave final approval for publication.

## Supporting information


**Figure S1.** Distinct and common wound responses across anatomical sites
**Figure S2.** Anatomical variations in dermal ECM

**Table S1.** Excel file of transcriptional signatures of anatomical sites in homeostasis (related to Figure 1)

**Table S2.** Excel file of development terms in all site‐specific signatures (related to Figures 1 and 4)

**Table S3.** Excel file of transcriptional signatures of anatomical sites during wound repair (related to Figures 2, 3., 4)

**Table S4.** Excel file of ECM terms in all site‐specific signatures (related to Figures 2 and S1)

**Table S5.** Excel file of wound‐induced changes to site‐specific signatures (related to Figure 4)

## Data Availability

The full RNA‐seq dataset is accessible at: GSE151850 (https://www.ncbi.nlm.nih.gov/geo/query/acc.cgi?acc=GSE151850).

## References

[1] McGrath JA , Uitto J . Anatomy and organization of human skin In Rook's Textbook of Dermatology (8th edn), Burns T , Breathnach S , Cox N , *et al* (eds). Wiley‐Blackwell: Oxford, 2010.

[2] Ferguson MW , O'Kane S . Scar‐free healing: from embryonic mechanisms to adult therapeutic intervention. Philos Trans R Soc Lond B Biol Sci 2004; 359 **:** 839–850. 15293811 10.1098/rstb.2004.1475 PMC1693363

[3] Ospelt C , Frank‐Bertoncelj M . Why location matters – site‐specific factors in rheumatic diseases. Nat Rev Rheumatol 2017; 13 **:** 433–442. 28615733 10.1038/nrrheum.2017.96

[4] Zwilling E . Interaction between ectoderm and mesoderm in duck‐chicken limb bud chimaeras. J Exp Zool 1959; 142 **:** 521–532. 13789035 10.1002/jez.1401420124

[5] Shaw TJ , Rognoni E . Dissecting fibroblast heterogeneity in health and fibrotic disease. Curr Rheumatol Rep 2020; 22 **:** 33. 32562113 10.1007/s11926-020-00903-w PMC7305072

[6] Driskell RR , Lichtenberger BM , Hoste E , *et al* Distinct fibroblast lineages determine dermal architecture in skin development and repair. Nature 2013; 504 **:** 277–281. 24336287 10.1038/nature12783 PMC3868929

[7] Rinkevich Y , Walmsley GG , Hu MS , *et al* Skin fibrosis. Identification and isolation of a dermal lineage with intrinsic fibrogenic potential. Science 2015; 348 **:** aaa2151. 25883361 10.1126/science.aaa2151 PMC5088503

[8] Chang HY , Chi JT , Dudoit S , *et al* Diversity, topographic differentiation, and positional memory in human fibroblasts. Proc Natl Acad Sci U S A 2002; 99 **:** 12877–12882. 12297622 10.1073/pnas.162488599 PMC130553

[9] Rinn JL , Bondre C , Gladstone HB , *et al* Anatomic demarcation by positional variation in fibroblast gene expression programs. PLoS Genet 2006; 2 **:** e119. 16895450 10.1371/journal.pgen.0020119 PMC1523235

[10] Kim D , Langmead B , Salzberg SL . HISAT: a fast spliced aligner with low memory requirements. Nat Methods 2015; 12 **:** 357–360. 25751142 10.1038/nmeth.3317 PMC4655817

[11] Langmead B , Salzberg SL . Fast gapped‐read alignment with Bowtie 2. Nat Methods 2012; 9 **:** 357–359. 22388286 10.1038/nmeth.1923 PMC3322381

[12] Li B , Dewey CN . RSEM: accurate transcript quantification from RNA‐Seq data with or without a reference genome. BMC Bioinform 2011; 12 **:** 323. 10.1186/1471-2105-12-323 PMC3163565 21816040

[13] Love MI , Huber W , Anders S . Moderated estimation of fold change and dispersion for RNA‐seq data with DESeq2. Genome Biol 2014; 15 **:** 550. 25516281 10.1186/s13059-014-0550-8 PMC4302049

[14] Saeed AI , Sharov V , White J , *et al* TM4: a free, open‐source system for microarray data management and analysis. Biotechniques 2003; 34 **:** 374–378. 12613259 10.2144/03342mt01

[15] Raudvere U , Kolberg L , Kuzmin I , *et al* g:Profiler: a web server for functional enrichment analysis and conversions of gene lists (2019 update). Nucleic Acids Res 2019; 47 **:** W191–W198. 31066453 10.1093/nar/gkz369 PMC6602461

[16] Kaukonen R , Jacquemet G , Hamidi H , *et al* Cell‐derived matrices for studying cell proliferation and directional migration in a complex 3D microenvironment. Nat Protoc 2017; 12 **:** 2376–2390. 29048422 10.1038/nprot.2017.107

[17] Schindelin J , Arganda‐Carreras I , Frise E , *et al* Fiji: an open‐source platform for biological‐image analysis. Nat Methods 2012; 9 **:** 676–682. 22743772 10.1038/nmeth.2019 PMC3855844

[18] Bickley SR , Logan MP . Regulatory modulation of the T‐box gene Tbx5 links development, evolution, and adaptation of the sternum. Proc Natl Acad Sci U S A 2014; 111 **:** 17917–17922. 25468972 10.1073/pnas.1409913111 PMC4273354

[19] Kawanishi T , Kaneko T , Moriyama Y , *et al* Modular development of the teleost trunk along the dorsoventral axis and zic1/zic4 as selector genes in the dorsal module. Development 2013; 140 **:** 1486–1496. 23462471 10.1242/dev.088567

[20] Trainor PA . Making headway: the roles of Hox genes and neural crest cells in craniofacial development. ScientificWorldJournal 2003; 3 **:** 240–264. 12806110 10.1100/tsw.2003.11 PMC5974867

[21] Favier B , Dolle P . Developmental functions of mammalian Hox genes. Mol Hum Reprod 1997; 3 **:** 115–131. 9239717 10.1093/molehr/3.2.115

[22] Whitfield ML , George LK , Grant GD , *et al* Common markers of proliferation. Nat Rev Cancer 2006; 6 **:** 99–106. 16491069 10.1038/nrc1802

[23] De Bellard ME , Rao Y , Bronner‐Fraser M . Dual function of Slit2 in repulsion and enhanced migration of trunk, but not vagal, neural crest cells. J Cell Biol 2003; 162 **:** 269–279. 12876276 10.1083/jcb.200301041 PMC2172792

[24] Miyahara T , Murai A , Tanaka T , *et al* Age‐related differences in human skin collagen: solubility in solvent, susceptibility to pepsin digestion, and the spectrum of the solubilized polymeric collagen molecules. J Gerontol 1982; 37 **:** 651–655. 6813368 10.1093/geronj/37.6.651

[25] Mostaço‐Guidolin L , Rosin NL , Hackett TL . Imaging collagen in scar tissue: developments in second harmonic generation microscopy for biomedical applications. Int J Mol Sci 2017; 18 **:** 1772. 10.3390/ijms18081772 PMC5578161 28809791

[26] Correa‐Gallegos D , Jiang D , Christ S , *et al* Patch repair of deep wounds by mobilized fascia. Nature 2019; 576 **:** 287–292. 31776510 10.1038/s41586-019-1794-y

[27] Londin ER , Mentzer L , Sirotkin HI . Churchill regulates cell movement and mesoderm specification by repressing Nodal signaling. BMC Dev Biol 2007; 7 **:** 120. 17980025 10.1186/1471-213X-7-120 PMC2180179

[28] Sweetman D , Wagstaff L , Cooper O , *et al* The migration of paraxial and lateral plate mesoderm cells emerging from the late primitive streak is controlled by different Wnt signals. BMC Dev Biol 2008; 8 **:** 63. 18541012 10.1186/1471-213X-8-63 PMC2435575

[29] Thomas T , Kurihara H , Yamagishi H , *et al* A signaling cascade involving endothelin‐1, dHAND and msx1 regulates development of neural‐crest‐derived branchial arch mesenchyme. Development 1998; 125 **:** 3005–3014. 9671575 10.1242/dev.125.16.3005

[30] Philippeos C , Telerman SB , Oules B , *et al* Spatial and single‐cell transcriptional profiling identifies functionally distinct human dermal fibroblast subpopulations. J Invest Dermatol 2018; 138 **:** 811–825. 29391249 10.1016/j.jid.2018.01.016 PMC5869055

[31] Walmsley GG , Rinkevich Y , Hu MS , *et al* Live fibroblast harvest reveals surface marker shift in vitro. Tissue Eng Part C Methods 2015; 21 **:** 314–321. 25275778 10.1089/ten.tec.2014.0118 PMC4346232

[32] Shaw TJ , Martin P . Wound repair at a glance. J Cell Sci 2009; 122 **:** 3209–3213. 19726630 10.1242/jcs.031187 PMC2736861

[33] Cooper L , Johnson C , Burslem F , *et al* Wound healing and inflammation genes revealed by array analysis of 'macrophageless' PU.1 null mice. Genome Biol 2005; 6 **:** R5. 15642097 10.1186/gb-2004-6-1-r5 PMC549066

[34] Shook BA , Wasko RR , Rivera‐Gonzalez GC , *et al* Myofibroblast proliferation and heterogeneity are supported by macrophages during skin repair. Science 2018; 362 **:** eaar2971. 30467144 10.1126/science.aar2971 PMC6684198

[35] Szabó A , Mayor R . Mechanisms of neural crest migration. Annu Rev Genet 2018; 52 **:** 43–63. 30476447 10.1146/annurev-genet-120417-031559

[36] Graham A . The neural crest. Curr Biol 2003; 13 **:** R381–R384. 12747846 10.1016/s0960-9822(03)00315-4

[37] Caplan AI . The extracellular matrix is instructive. Bioessays 1986; 5 **:** 129–132. 3566725 10.1002/bies.950050309

[38] Nash LG , Phillips MN , Nicholson H , *et al* Skin ligaments: regional distribution and variation in morphology. Clin Anat 2004; 17 **:** 287–293. 15108331 10.1002/ca.10203

[39] Arda O , Göksügür N , Tüzün Y . Basic histological structure and functions of facial skin. Clin Dermatol 2014; 32 **:** 3–13. 24314373 10.1016/j.clindermatol.2013.05.021

[40] Martin P , Parkhurst SM . Parallels between tissue repair and embryo morphogenesis. Development 2004; 131 **:** 3021–3034. 15197160 10.1242/dev.01253

[41] Tran TH , Jarrell A , Zentner GE , *et al* Role of canonical Wnt signaling/β‐catenin via Dermo1 in cranial dermal cell development. Development 2010; 137 **:** 3973–3984. 20980404 10.1242/dev.056473 PMC2976281

[42] Ohtola J , Myers J , Akhtar‐Zaidi B , *et al* β‐Catenin has sequential roles in the survival and specification of ventral dermis. Development 2008; 135 **:** 2321–2329. 18539925 10.1242/dev.021170 PMC2732188

[43] Atit R , Sgaier SK , Mohamed OA , *et al* Beta‐catenin activation is necessary and sufficient to specify the dorsal dermal fate in the mouse. Dev Biol 2006; 296 **:** 164–176. 16730693 10.1016/j.ydbio.2006.04.449

[44] Tong PL , Roediger B , Kolesnikoff N , *et al* The skin immune atlas: three‐dimensional analysis of cutaneous leukocyte subsets by multiphoton microscopy. J Invest Dermatol 2015; 135 **:** 84–93. 25007044 10.1038/jid.2014.289 PMC4268113

[45] Grice EA , Segre JA . The skin microbiome. Nat Rev Microbiol 2011; 9 **:** 244–253. 21407241 10.1038/nrmicro2537 PMC3535073

[46] Stephens P , Davies KJ , Occleston N , *et al* Skin and oral fibroblasts exhibit phenotypic differences in extracellular matrix reorganization and matrix metalloproteinase activity. Br J Dermatol 2001; 144 **:** 229–237. 11251552 10.1046/j.1365-2133.2001.04006.x

[47] Iglesias‐Bartolome R , Uchiyama A , Molinolo AA , *et al* Transcriptional signature primes human oral mucosa for rapid wound healing. Sci Transl Med 2018; 10 **:** eaap8798. 30045979 10.1126/scitranslmed.aap8798 PMC6598699

[48] Peake MA , Caley M , Giles PJ , *et al* Identification of a transcriptional signature for the wound healing continuum. Wound Repair Regen 2014; 22 **:** 399–405. 24844339 10.1111/wrr.12170 PMC4230470

