# Supplementary material for: A developmental basis for the anatomical diversity of dermis in homeostasis and wound repair
Source: J Pathol. 2020 Dec 4;253(3):315–25. doi: 10.1002/path.5589 (PMC7898902; doi:10.1002/path.5589)
Supplement: Supplementary file 1 — Figure S1. Distinct and common wound responses across anatomical sites Figure S2. Anatomical variations in dermal ECM [file PATH-253-315-s001.docx]

**A developmental basis for the anatomical diversity of dermis in homeostasis and wound repair**

I Usansky *et al. J Pathol* DOI: 10.1002/path.5589

**Supplementary Figures S1,S2**


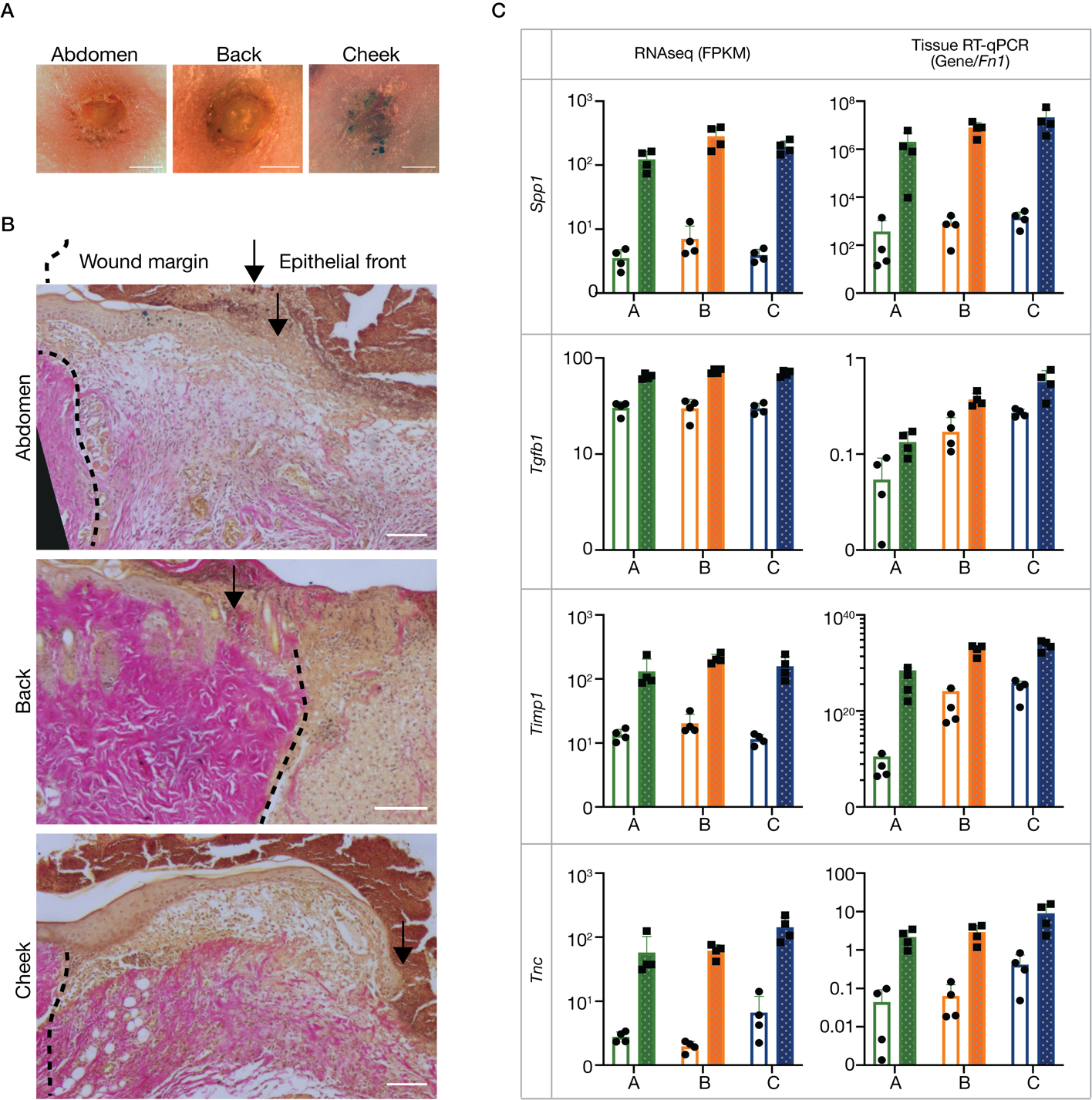


**Figure S1. Distinct and common wound responses across anatomical sites.**

(A) Gross and (B) histological analyses (Haematoxylin Van Gieson) of dermis illustrating anatomically distinct responses to tissue damage. Scale bars: A, 1 mm; B, 200 µm. (C) RNA expression of highly significant wound-induced genes common to all sites, as determined by RNA-seq (FPKM, left column), and RT-qPCR (right column). RT-qPCR bar charts show relative quantification, where concentrations were determined against a standard curve, and results normalised to *Fn1*. Plotted points are skin/wound isolations from 4 mice per anatomical

site. Bars/error bars = mean ± SD.


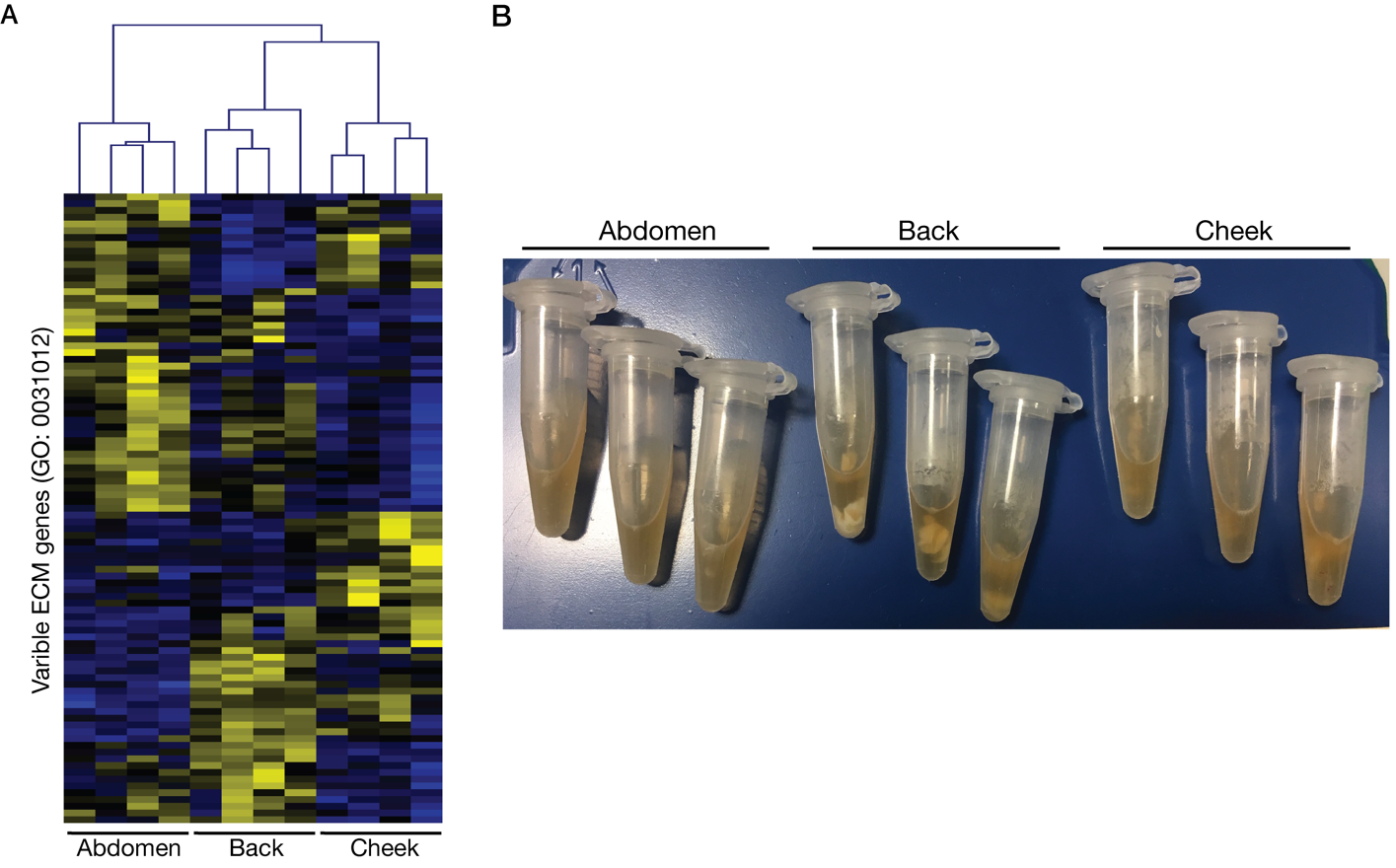


**Figure S2. Anatomical variations in dermal ECM.**

(A) Heatmap and dendrogram displaying the hierarchical clustering of samples based on the expression of the 93 significantly altered (padj<0.05) genes assigned to the ECM GO term (GO:0031012). (B) Photograph illustrating the extent of enzymatic digestion (after 1 h at 37 oC) of 4 mm biopsy punches of skin from abdomen, back, and cheek, with the back samples still showing clearly intact/undigested tissue.
